# Correlation between Body Mass Index and Cognitive Function in Patients with Atrial Fibrillation

**DOI:** 10.1155/2022/6025732

**Published:** 2022-12-14

**Authors:** Li Guo, Ping Yin, Xiangting Li, Xin Wang, Jing Xue, Wenqing Wang, Dongmei Song, Guomei Xu, Miaomiao Shang, Shuai Liu, Yuanyuan Chen, Qingyun Zhang, Dandan Sun

**Affiliations:** Affiliated Hospital of Jining Medical University, Guhuai Road 89#, Rencheng, Jining, Shandong, China

## Abstract

**Background:**

Evidence regarding the relationship between body mass index (BMI) and cognitive function was limited. Therefore, the objective of this research is to investigate whether BMI is independently related to cognitive function in Chinese patients with atrial fibrillation after adjusting for other covariates.

**Methods:**

The present study is a cross-sectional study. A total of 281 patients with atrial fibrillation who were hospitalized at the Affiliated Hospital of Jining Medical University in Shandong Province from January 2021 to November 2021 were included in the study. The target independent variable and the dependent variable were BMI and cognitive function in patients with atrial fibrillation, respectively. The patients' general information, BMI, past history, medication history, and other disease-related data were collected. The Montreal cognitive assessment scale (MoCA) was used to evaluate cognitive function.

**Results:**

A total of 244 patients with atrial fibrillation were collected in this study, with an average age of (67.28 ± 10.33) years, of whom 55.3% were male. The average BMI was (25.33 ± 4.27) kg/m^2^, and the average cognitive function score was (19.25 ± 6.88) points. The results of the smooth curve fitting and threshold effect tests showed that there was a curve correlation between BMI and cognitive function score, and its inflection point was 24.56 kg/m^2^. To the left of the inflection point, the relationship was significant; the effect size and the confidence interval were 0.43 and 0.01–0.85, respectively. To the right of the inflection point, there was no significant correlation between BMI and cognitive function (*P*=0.152).

**Conclusion:**

When BMI is lower than 24.56 kg/m^2^, the cognitive function score increases by 0.43 points for each unit increase in BMI in patients with atrial fibrillation. An increase in BMI at this time is a protective factor for cognitive function. Within the normal range of BMI, the higher the BMI in atrial fibrillation patients, the higher the cognitive function score. We encourage atrial fibrillation patients with normal BMI to maintain their current weight.

## 1. Introduction

Atrial fibrillation is a supraventricular tachyarrhythmia with uncoordinated atrial electrical activation and ineffective atrial contractions [1]. The lifetime risk of atrial fibrillation is 1 in 4 and increases to 1 in 3 in people over 55 years of age [2–5]. Atrial fibrillation will lead to impaired cognitive function in patients. Alonso et al. [6] showed that the incidence of dementia and mild cognitive impairment in patients with atrial fibrillation was 40%, and the risks was 2.25 times and 1.28 times that of the normal population, respectively. In addition, Chen et al. [7] showed that atrial fibrillation is a risk factor for dementia and cognitive impairment independent of stroke. Cognitive decline or even dementia in patients with atrial fibrillation will seriously reduce the quality of life of patients and increase the burden on family caregivers [8, 9]. Studies have shown that obesity may lead to cognitive decline [10–12]. But there are also studies suggesting that obesity may be a protective factor for cognitive function [13, 14]. At present, no studies have explored the relationship between obesity and cognitive function in patients with atrial fibrillation, so the relationship between the two is unclear. If the correlation between body mass index (BMI) and cognitive function in patients with atrial fibrillation can be clarified, it can provide a basis for the prevention of cognitive dysfunction in patients with atrial fibrillation. Therefore, our study aimed to describe the association between BMI and cognitive impairment in patients with atrial fibrillation. The results of this study can help clinicians determine the target value of weight control in patients with atrial fibrillation, and provide evidence for improving the cognitive function of patients with atrial fibrillation.

## 2. Methods

### 2.1. Study Design

This study is a cross-sectional study. The purpose of this study was to explore the relationship between BMI and cognitive function scores in patients with atrial fibrillation. The independent variable was the BMI of patients with atrial fibrillation. The dependent variable was cognitive function scores in patients with atrial fibrillation.

### 2.2. Participants

**Figure 1:**
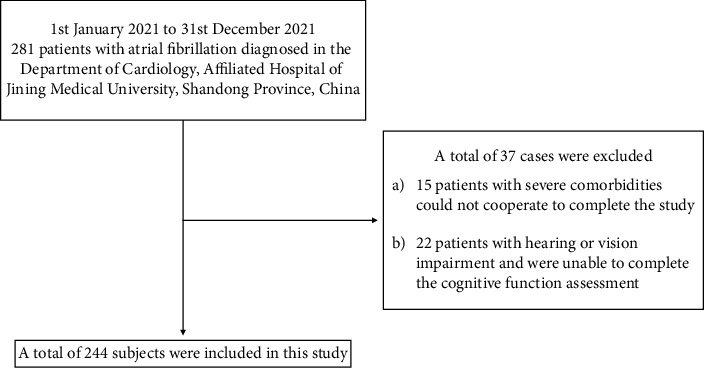
Inclusion/exclusion criteria.

The convenience sampling method was used to recruit patients with atrial fibrillation who were hospitalized in the Cardiology Department of a tertiary hospital in Shandong Province from January 2021 to November 2021 (Figure 1). Inclusion criteria were: (1) age ≥18 years; (2) diagnosis of atrial fibrillation after 12-lead ECG and 24 hour Holter monitoring according to the diagnostic criteria of the 2020 ECS/EACTS Guidelines for the Diagnosis and Management of Atrial Fibrillation [15]; (3) signing the informed consent form. The exclusion criteria were: (1) patients with severe comorbidities could not cooperate to complete the study; (2) patients with hearing or vision impairment were unable to complete the cognitive function assessment. This study was approved by the Ethics Committee of the Affiliated Hospital of Jining Medical College (ethics batch number: 2020C009).

### 2.3. Variables

The covariates collected in this study included two parts: general patient data and clinical data. (1) General information of patients: including the patients' age, sex, marital status, education level, and living conditions. (2) Clinical data of the patients: including the patients' smoking history, drinking history, medication use within 1 month before admission, coronary heart disease history, history of myocardial infarction, history of cerebral infarction, diabetes, hypertension, valvular disease, hyperlipidemia, history of heart failure, systolic blood pressure (SBP), diastolic blood pressure (DBP), D-dimer, left ventricular blood fraction (LVEF), creatinine, free thyroxine, free triiodothyronine, and left atrial diameter.

### 2.4. Research Tool: Montreal Cognitive Assessment Scale (MoCA)

The scale measures the following aspects: (1) short-term memory; (2) visuospatial ability; (3) executive ability; (4) attention, numeracy, and working memory; (5) language; and (6) orientation. We will add 1 point to the score for less than 12 years of education to correct for the effect of educational attainment (if the total score is less than 30 points) [16, 17]. The full score of MoCA is 30 points. The higher the score, the better the patient's cognitive function. When the score ≥26 points, the patient's cognitive function is normal; otherwise, the patient will be recognized as having cognitive dysfunction. The evaluation process takes approximately 10 minutes. The MoCA had a high sensitivity of 0.94 [18].

### 2.5. Data Collection

Patients who met the inclusion criteria signed the informed consent form after admission and completed the evaluation using the Montreal Cognitive Function Assessment Scale within 24 hours. The cognitive function assessment was performed by specially trained nurses. In the process of cognitive function measurement, the patient was guaranteed to be in a calm state. If the patient has a sudden change in condition, the measurement is discontinued. The nurses collected the general information and disease-related information of the patients. The measurement of systolic and diastolic blood pressure uses a unified measurement standard. The patients were in a supine position when blood pressure was measured. We properly use a small towel to raise the upper arm so that it is at the same level as the heart. We measured blood pressure three times for the patients, and took the average of the three measurements as the final blood pressure value. During height and weight measurement, patients took off their coat and shoes and kept only light clothing. BMI was defined as weight in kilograms divided by height in meters squared (kg/m^2^). In the morning of the next day after admission, the nurses collected the fasting blood of the patients for the collection of laboratory indicators. Cardiac ultrasonography was performed by a qualified sonographer, and the patients' left ventricular ejection fraction and left atrial diameter were collected.

### 2.6. Statistical Analysis

Continuous variables were tested for normality using the K–S method. Normally distributed variables were expressed as mean ± standard deviation (*x* ± *s*), and one-way ANOVA was used. Non-normally distributed variables were expressed as medians (minimum, maximum), and the Kruskal-Wallis H test was used. Categorical variables were expressed as frequencies or percentages (%) using the *χ* test. First, univariate analysis was performed to compare the baseline characteristics of patients with atrial fibrillation in different BMI groups. Univariate analysis was performed on the occurrence of cognitive impairment. The relationship between BMI levels and cognitive impairment in different models was then analyzed. We built a total of 3 models. Model 1: Rough model without adjustment for any covariates; Model 2: Adjusted for sociodemographic data only; Model 3: Model adjusted for all confounders. We addressed the non-linear relationship between BMI and the occurrence of cognitive impairment using threshold effect tests and smooth curve fitting. All the analyses were performed with the statistical software packages R (https://www.R-project.org, the R Foundation) and EmpowerStats (https://www.empowerstats.com, X&Y Solutions, Inc, Boston, MA). *P* values less than 0.05 (two-sided) were considered statistically significant.

## 3. Results

### 3.1. Basic Characteristics of the Research Subjects (Table 1)

A total of 244 patients with atrial fibrillation were collected for this study, with an average age of (67.28 ± 10.33) years, of whom 55.3% were male. The average BMI was 25.33 ± 4.27 kg/m^2^. The average cognitive function score of the research subjects was 19.25 ± 6.88 points. The incidence of cognitive dysfunction was 80.33%. We divided 244 subjects into three groups equally: the BMI ≤ 23.3 kg/m^2^ group, the 23.3 < BMI ≤ 26.6 kg/m^2^ group, and the BMI >26.6 kg/m^2^ group. The number of patients in each group was essentially the same. In different groups, there were significant differences in whether or not people took aspirin for more than 1 month, diabetes, hypertension, valvular disease, hyperlipidemia, coronary heart disease, heart failure, SBP, free thyroxine, and other indicators before medication (*P* < 0.05).

### 3.2. Univariate Analysis Results of Cognitive Dysfunction in Patients with Atrial Fibrillation (Table 2)

Univariate analysis showed that age (*F* = 1.522, *P*=0.025), sex (*F* = 14.440, *P* < 0.001), smoking history (*F* = 3.848, *P*=0.010), drinking history (*F* = 4.544, *P*=0.012), educational level (*F* = 17.404, *P* < 0.001), and hyperlipidemia (*F* = 7.795, *P*=0.006) were significantly associated with the occurrence of cognitive impairment in patients with atrial fibrillation.

### 3.3. Multiple Regression Analysis Results (Table 3)

In this study, three models were constructed to explore whether there was a linear correlation between BMI and cognitive function scores in patients with atrial fibrillation. Model-based effect sizes were interpreted as the change in the MoCA score for each additional unit of BMI value. Only in Model 1, without adjustment for other variables, was BMI positively associated with cognitive function scores. In model 2 and model 3, the correlation between BMI and the cognitive function score was not statistically different. There was no linear correlation between BMI and cognitive function scores in patients with atrial fibrillation.

### 3.4. The Nonlinear Relationship between BMI and Cognitive Function Score (Table 4, Figure 2)

**Figure 2:**
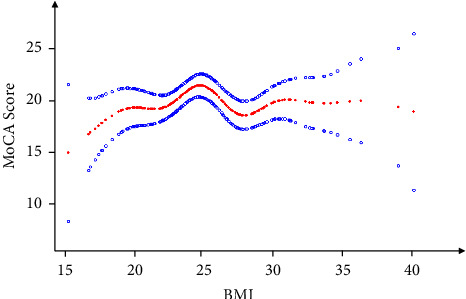
Association between BMI and cognitive function of atrial fibrillation. A threshold and a non-linear relationship between BMI and MoCA score were found in a generalized additive model. The solid red line represents the smooth curve fit between variables. Blue bands represent the 95% confidence interval from the fit. Models were adjusted for age, sex, smoking or not, alcohol consumption, marital status, degree of education, living condition, aspirin, warfarin, history of myocardial infarction, cerebral infarction history, diabetes mellitus, hypertension, valvular disease, hyperlipidemia, coronary artery disease, heart failure, SBP, DBP, *D*-dimer, creatinine, FT4, FT3, left atrial diameter.

In the non-linear relationship analysis of BMI and cognitive function scores, smooth curve fitting and threshold effect test results showed that there was a curvilinear relationship between the two. Using multiple regression and recursive algorithms, the inflection point was calculated to be 24.56 kg/m^2^. To the left of the inflection point, the relationship was significant, with effect sizes and 95% CI of 0.43 and 0.01–0.85, respectively. To the right of the inflection point, there was no significant correlation between the two (*P*=0.152). A second inflection point can be observed in Figure 2, but we do not find statistical significance.

## 4. Discussion

The results of this study showed a non-linear relationship between BMI and cognitive function scores in patients with atrial fibrillation after adjustment for other covariates. The turning point of the relationship between the two is 24.56 kg/m^2^ and on the right side of the inflection point, there is no significant correlation between the two. On the left side of the inflection point, cognitive function scores increased by 0.43 points for every one-unit increase in BMI in patients with atrial fibrillation.

Previous studies have shown that obesity is a risk factor for cognitive impairment [10, 19, 20]. In children and adolescents, obesity leads to impaired cognitive function [21], and middle age is also an important factor leading to cognitive decline [22]. Meta-analysis results showed that executive function, short-term memory, and other dimensions of cognitive function are negatively correlated with BMI [23, 24]. The possible reasons for the decline of cognitive function caused by obesity are: (1) Obesity leads to neurological atrophy in patients. For example, the increase in the BMI index is related to the decrease of the brain volume, gray matter atrophy, and white matter decrease in patients [25]; (2) obesity leads to changes in neural activity. Elevated BMI led to a decrease in regional blood flow in the frontal lobes of patients. Activity in cortical regions related to episodic memory (hippocampus, angular gyrus, and dorsolateral prefrontal cortex) is also associated with obesity and insulin resistance [26]; (3) obesity leads to brain aging, and middle-aged obesity leads to the most severe brain aging. In obese patients, adiponectin levels are reduced [27]. Adiponectin prevents inflammation promotes cell proliferation and supports the upregulation of energy metabolism and other adipokines [28].

However, there is still an “obesity paradox,” that is, obesity is a protective factor for cognitive function. Ely et al. [29] explored the relationship between cognitive function and obesity in patients with heart failure. Studies have shown that non-obese patients have a higher risk of cognitive dysfunction than obese patients. Qizilbash et al. [30] conducted a study on the relationship between BMI and the occurrence of dementia. The study included nearly 2 million subjects. The results showed that compared with healthy weight people, people with lower body weight (BMI <20 kg/m^2^) suffered from the risk of dementia was 34% higher (95% CI 29–38). The study followed the population for 15 years, and the obesity paradox persists. In addition, the incidence of dementia continued to decline with each grade-level increase in BMI. People who were very obese (BMI >40 kg/m^2^) had a 29% lower risk of dementia compared to people of healthy weight. The reasons for the existence of the obesity paradox may include diet, exercise, genetics, and other factors, but the specific mechanism is not fully understood.

Different from the results of the above two aspects, the results of this study found a specific threshold for the impact of BMI on cognitive function. When the BMI value of patients with atrial fibrillation was less than 24.56 kg/m^2^, the cognitive function score increased by 0.43 points for each unit of increase in the BMI of patients with atrial fibrillation. An increase in BMI at this time is a protective factor for cognitive function, which is consistent with the findings of Qizilbash et al. [30] discussed above. However, when BMI was greater than 24.56 kg/m^2^, the relationship between BMI and cognitive function was not significant, which was different from the results of the previous studies described above. The reason may be that the population of this study is patients with atrial fibrillation, and the mechanism of cognitive decline in patients with atrial fibrillation is more complex, including blood hypercoagulability, the formation of inflammatory factors, decreased cerebral blood perfusion, and genetic changes [31]. The population of this study differs from previous studies, which may explain the existence of a threshold between BMI and cognitive function scores in patients with atrial fibrillation. For the Asian population, the standard BMI range is 18.5–23.9 kg/m^2^, and the threshold obtained in this study was 24.56 kg/m^2^. Within the normal range of BMI, the higher the BMI, the higher the cognitive function score in patients with atrial fibrillation.

Decreased cognitive dysfunction in patients with atrial fibrillation leads to a decline in the quality of life of patients and seriously increases the burden on family caregivers, so its prevention and management are crucial. From the perspective of the relationship between BMI index and cognitive function score, this study explored the nonlinear relationship between the two after adjusting for covariates, which can provide an important basis for the prevention of cognitive dysfunction based on weight management. However, this study also has certain limitations. (1) This study only included a single-center AF patient population in southwestern Shandong Province, so the results may be different in other races, which may have some limitations in the external applicability of the results; (2) This study was a cross-sectional study, and no follow-up of patients was carried out. In the future, long-termfollow-up and tracking of patients' BMI and cognitive function level can be carried out to further explore the longitudinal relationship between the two; (3) Obesity can be reflected by many indicators including BMI, waist circumference, abdominal circumference, and waist-to-hip ratio. Only BMI was used in this study. Regarding the relationship between obesity and cognitive function in patients with atrial fibrillation, more comprehensive indicators can be used to reflect the degree of obesity in the future.

## 5. Conclusions

The results of this study showed that when the BMI index of patients with atrial fibrillation was lower than 24.56 kg/m^2^, the cognitive function score increased by 0.43 points for each unit of increase in BMI of patients with atrial fibrillation. The increase of BMI at this time was a protective factor for cognitive function. However, the relationship between the two was not significant when the BMI exceeded 24.56 kg/m^2^. Within the normal range of BMI, the higher the BMI of patients with atrial fibrillation, the higher their cognitive function scores. We encourage atrial fibrillation patients with a normal BMI to maintain their current weight. However, for patients with a BMI of more than 24.56 kg/m^2^, it is necessary to consider multiple clinical perspectives to guide patients and maintain a healthy weight.

## Figures and Tables

**Table 1:** Baseline characteristics of participants.

BMI	*T*1	*T*2	*T*3	*P* value
*N*	81	82	81	
Age (year)	68.87 ± 11.33	66.67 ± 10.35	66.01 ± 9.81	0.173
Sex, *n* (%)				0.118
Male	41 (50.6)	42 (51.2)	52 (64.2)	
Female	40 (49.4)	40 (48.8)	29 (35.8)	
Smoking or not, *n* (%)				0.843
Current smoker	49 (60.5)	50 (61.0)	44 (54.3)	
Nonsmoker	19 (23.5)	17 (20.7)	19 (23.5)	
Quit	13 (16.0)	15 (18.3)	18 (22.2)	
Alcohol consumption, *n* (%)				0.429
Current drinker	58 (71.6)	60 (73.2)	54 (66.7)	
Nondrinker	14 (17.3)	18 (22.0)	18 (22.2)	
Quit	9 (11.1)	4 (4.8)	9 (11.1)	
Marital status, *n* (%)				0.687
Unmarried/divorce	1 (1.3)	1 (1.2)	1 (1.2)	
Married	69 (85.2)	69 (84.1)	72 (88.8)	
Widowed	11 (13.6)	12 (14.6)	8 (9.9)	
Degree of education, *n* (%)				0.104
Illiterate	33 (40.7)	29 (35.3)	17 (21.0)	
Primary and junior high school	34 (42.0)	39 (47.6)	42 (51.9)	
High school	7 (8.6)	9 (11.0)	15 (18.5)	
Undergraduate and above	6 (7.4)	5 (6.1)	7 (8.6)	
Living condition, *n* (%)				0.396
Live alone	3 (3.7)	8 (9.9)	6 (7.4)	
Living with a spouse	64 (79.0)	65 (79.3)	66 (81.5)	
Living with children	11 (13.6)	9 (11.0)	8 (9.9)	
Other	3 (3.7)	0 (0.0)	1 (1.2)	
Aspirin, *n* (%)				0.021
No	55 (67.9)	59 (72.0)	43 (53.1)	
Yes	26 (32.1)	23 (28.0)	38 (46.9)	
Warfarin, *n* (%)				0.889
No	72 (88.9)	71 (86.0)	71 (87.4)	
Yes	9 (11.1)	11 (14.0)	10 (12.6)	
History of myocardial infarction, *n* (%)				0.138
No	74 (91.4)	79 (96.3)	72 (88.9)	
Yes	7 (8.6)	3 (3.7)	9 (11.1)	
Cerebral infarction history, *n* (%)				0.344
No	69 (85.1)	72 (87.8)	64 (79.1)	
Yes	12 (14.9)	10 (12.2)	17 (20.9)	
Diabetes mellitus, *n* (%)				<0.001
No	72 (88.9)	56 (68.3)	54 (66.7)	
Yes	9 (11.1)	26 (31.7)	27 (33.3)	
Hypertension, *n* (%)				<0.001
No	50 (61.7)	36 (43.9)	27 (33.3)	
Yes	31 (38.3)	46 (56.1)	54 (66.7)	
Valvular disease, *n* (%)				<0.001
No	43 (53.1)	51 (62.2)	68 (83.9)	
Yes	38 (46.9)	31 (37.8)	13 (16.1)	
Hyperlipidemia, *n* (%)				0.253
No	80 (98.9)	79 (96.3)	76 (93.8)	
Yes	1 (1.1)	3 (3.7)	5 (6.2)	
Coronary artery disease, *n* (%)				0.018
No	23 (28.4)	16 (19.5)	9 (11.1)	
Yes	58 (71.6)	66 (80.5)	72 (88.9)	
Heart failure, *n* (%)				0.007
No	25 (30.9)	44 (53.7)	40 (49.4)	
Yes	56 (69.1)	38 (46.3)	41 (50.6)	
SBP (mmHg)	120.67 ± 21.04	128.28 ± 18.62	131.36 ± 23.84	0.003
DBP (mmHg)	73.06 ± 15.01	79.01 ± 13.37	81.93 ± 15.32	<0.001
D-dimer (mg/L)	1.25 ± 1.76	0.75 ± 1.36	0.88 ± 1.77	0.133
LVEF (%)	47.77 ± 13.07	50.42 ± 12.88	52.12 ± 9.65	0.063
Creatinine (umol/L)	77.69 ± 39.96	72.15 ± 20.02	74.72 ± 28.10	0.514
FT4 (pmol/L)	21.24 ± 14.22	17.81 ± 3.89	17.07 ± 4.92	0.009
FT3 (pmol/L)	5.30 ± 4.91	4.54 ± 2.66	4.58 ± 1.14	0.264
Left atrial diameter	48.21 ± 11.10	46.48 ± 9.01	47.23 ± 7.32	0.490

SBP, systolic blood pressure; DBP, diastolic blood pressure; FT3, free triiodothyronine; FT4, free thyroxine.

**Table 2:** Univariate analysis of cognitive dysfunction in patients with atrial fibrillation.

Covariate	*X* ± *S*/*N* (%)	*F*	*P* value
Age (year)	67.28 ± 10.33	1.522	0.025
Sex, *n* (%)			
Male	135 (55.3)	14.440	<0.001
Female	109 (44.7)		
Smoking or not, *n* (%)		3.848	0.010
Current smoker	53 (21.5)		
Nonsmoker	145 (58.9)		
Quit	46 (19.6)		
Alcohol consumption, *n* (%)		4.544	0.012
Current drinker	46 (18.7)		
Nondrinker	176 (71.5)		
Quit	22 (8.9)		
Marital status, *n* (%)		0.264	0.851
Unmarried/divorce	2 (0.8)		
Married	211 (85.8)		
Widowed	31 (12.6)		
Degree of education, *n* (%)		17.404	<0.001
Illiterate	81 (32.9)		
Primary and junior high school	113 (45.9)		
High school	32 (13.0)		
Undergraduate and above	18 (7.3)		
Living condition, *n* (%)		0.456	0.713
Live alone	15 (6.1)		
Living with a spouse	198 (80.5)		
Living with children	28 (11.4)		
Other	3 (11.2)		
Aspirin, *n* (%)		1.704	0.193
No	157 (63.8)		
Yes	87 (35.4)		
Warfarin, *n* (%)		1.301	0.255
No	215 (87.4)		
Yes	29 (11.8)		
History of myocardial infarction, *n* (%)		0.195	0.659
No	225 (92.2)		
Yes	19 (7.8)		
Cerebral infarction history, *n* (%)		0.209	0.648
No	203 (83.2)		
Yes	41 (16.8)		
Diabetes mellitus, *n* (%)		0.199	0.656
No	184 (75.4)		
Yes	60 (24.6)		
Hypertension, *n* (%)		0.141	0.708
No	111 (45.5)		
Yes	133 (54.5)		
Valvular disease, *n* (%)		1.642	0.201
No	164 (67.2)		
Yes	80 (32.8)		
Hyperlipidemia, *n* (%)		7.795	0.006
No	235 (96.3)		
Yes	9 (3.7)		
Coronary artery disease, *n* (%)		0.395	0.530
No	48 (19.7)		
Yes	196 (80.3)		
Heart failure, *n* (%)		1.179	0.279
No	105 (43.0)		
Yes	139 (57.0)		
SBP (mmHg)	126.80 ± 21.74	0.864	0.770
DBP (mmHg)	78.04 ± 15.09	0.906	0.672
*D*-dimer (mg/L)	0.92 ± 0.48	0.764	0.925
LVEF (%)	50.42 ± 11.77	0.875	0.705
Creatinine (umol/L)	74.30 ± 30.21	0.967	0.576
FT4 (pmol/L)	18.77 ± 9.33	0.950	0.610
FT3 (pmol/L)	4.84 ± 3.35	0.868	0.750
Left atrial diameter	47.34 ± 9.31	1.016	0.454

SBP, systolic blood pressure; DBP, diastolic blood pressure; FT3, free triiodothyronine; FT4, free thyroxine.

**Table 3:** Relationship between BMI and cognitive function in different models.

Variable	Crude model		Adjust 1		Adjust 2	
	*β* (95%CI)	*P*	*β* (95%CI)	*P*	*β* (95%CI)	*P*
BMI	0.35 (0.16, 0.54)	<0.001	0.00 (−0.18, 0.18)	0.993	0.15 (−0.25, 0.54)	0.467
BMI						
*T*1	Reference		Reference		Reference	
*T*2	2.55 (0.57, 4.53)	0.012	0.81 (−1.39, 3.00)	0.412	0.63 (−1.57, 2.83)	0.575
*T*3	3.29 (1.31, 5.26)	0.001	−2.22 (−5.59, 1.15)	0.199	−2.42 (−5.77, 0.93)	0.159

Crude model adjusted for none. Adjust I model adjusted for age and sex. Adjust II model adjusted for age, sex, smoking or not, alcohol consumption, marital status, degree of education, living condition, aspirin, warfarin, history of myocardial infarction, cerebral infarction history, diabetes mellitus, hypertension, valvular disease, hyperlipidemia, coronary artery disease, heart failure, SBP, DBP, *D*-dimer, creatinine, FT4, FT3, left atrial diameter.

**Table 4:** Results of BMI and cognitive function using two piecewise linear regression.

Inflection BMI (kg/m^2^)		Point of Effect size (kg/m^2^)	95% CI	P value
<24.56		0.43	(0.01, 0.85)	0.044
≥24.56		-0.19	(-0.44, 0.07)	0.152

Effect: cognitive function; cause: BMI (body mass index). Adjusted for age, sex, smoking or not, alcohol consumption, marital status, degree of education, living condition, aspirin, warfarin, history of myocardial infarction, cerebral infarction history, siabetes mellitus, hypertension, valvular disease, hyperlipidemia, coronary artery disease, heart failure, SBP, DBP, *D*-dimer, creatinine, FT4, FT3, left atrial diameter.

## Data Availability

The data used to support the findings of this study are available from the corresponding author upon request.
